# Effect of Surface Hydrophobicity on the Adsorption of a Pilus-Derived Adhesin-like Peptide

**DOI:** 10.1021/acs.langmuir.2c01016

**Published:** 2022-07-23

**Authors:** Yu Yang, Jingyuan Huang, Daniel Dornbusch, Guido Grundmeier, Karim Fahmy, Adrian Keller, David L. Cheung

**Affiliations:** † Technical and Macromolecular Chemistry, Paderborn University, Warburger Str. 100, 33098 Paderborn, Germany; ‡ Institute of Resource Ecology, Biophysics Department, Helmholtz-Zentrum Dresden-Rossendorf, Bautzner Landstrasse 400, 01328 Dresden, Germany; § Center for Molecular and Cellular Bioengineering, Technische Universität Dresden, 01062 Dresden, Germany; ∥ School of Chemistry, National University of Ireland Galway, Galway H91 TK33, Ireland

## Abstract

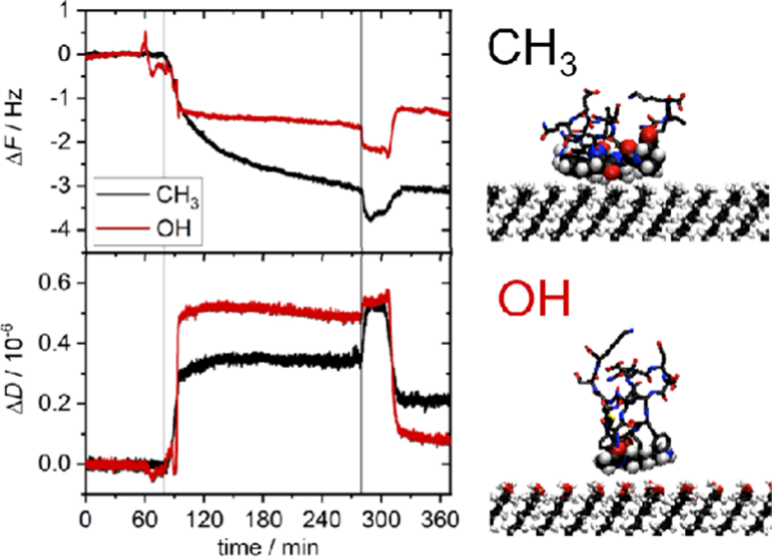

Bacterial colonization of abiotic surfaces such as those of medical implants, membrane filters, and everyday household items is a process of tremendous importance for public health. Bacteria use adhesive cell surface structures called adhesins to establish contact with abiotic surfaces. Among them, protein filaments called type IV pili are particularly important and found in many Gram-negative pathogens such as *Pseudomonas aeruginosa*. Understanding the interaction of such adhesin proteins with different abiotic surfaces at the molecular level thus represents a fundamental prerequisite for impeding bacterial colonization and preventing the spread of infectious diseases. In this work, we investigate the interaction of a synthetic adhesin-like peptide, PAK128-144ox, derived from the type IV pilus of *P. aeruginosa* with hydrophilic and hydrophobic self-assembled monolayers (SAMs). Using a combination of molecular dynamics (MD) simulations, quartz crystal microbalance with dissipation monitoring (QCM-D), and spectroscopic investigations, we find that PAK128-144ox has a higher affinity for hydrophobic than for hydrophilic surfaces. Additionally, PAK128-144ox adsorption on the hydrophobic SAM is furthermore accompanied by a strong increase in α-helix content. Our results show a clear influence of surface hydrophobicity and further indicate that PAK128-144ox adsorption on the hydrophobic surface is enthalpically favored, while on the hydrophilic surface, entropic contributions are more significant. However, our spectroscopic investigations also suggest aggregation of the peptide under the employed experimental conditions, which is not considered in the MD simulations and should be addressed in more detail in future studies.

## Introduction

Microbial colonization of abiotic surfaces and subsequent biofilm formation are ubiquitous phenomena with severe implications for various areas of technology and human health, ranging from microbial corrosion^1^ to drinking water purification^2^ and distribution^3^ to implant-associated infections.^4^ Initial contact between bacterial and fungal cells and a biotic or abiotic surface is facilitated by protein- or polysaccharide-based cell surface structures called adhesins.^5,6^ Dynamic multiprotein fibers called pili represent a particularly important class of adhesins that protrude from the surfaces of Gram-positive and Gram-negative bacteria. They are involved in surface attachment and subsequent motility^5^ and often contribute to pathogenicity.^7^ Type IV pili are a particularly widespread class of virulence-associated pili and found in many pathogenic bacteria, including *Neisseria gonorrhoeae*, *Neisseria meningitides*, *Vibrio cholera*, and *Pseudomonas aeruginosa*.^8^ For the latter, it was shown that the C-terminal D-region of the major pilin protein not only facilitates binding to the asialo-GM1 receptor of epithelial cells but is involved also in its attachment to abiotic surfaces such as stainless steel and various polymers.^9^ Most intriguingly, it was further demonstrated that *P. aeruginosa* adhesion to stainless steel surfaces could efficiently be inhibited by pre-adsorption of a synthetic peptide analogue of the receptor-binding pilin sequence.^9^ This synthetic peptide termed PAK128-144ox has the sequence Ac-KCTSDQDEQFIPKGCSK-OH and adopts a loop conformation stabilized by a disulfide bridge between the C- and N-terminal cysteines. It furthermore features three positively charged, three negatively charged, and four hydrophobic amino acid residues, allowing it to adsorb to both hydrophobic and hydrophilic surfaces. Consequently, PAK128-144ox was found to adsorb also at other abiotic surfaces, namely, Au, SiO_2_, and oxidized Ti, albeit with strongly different affinities.^10^

In this work, we aim to elucidate the role of one important surface property, the surface hydrophobicity, on PAK128-144ox adsorption at a molecular level. To this end, we combined molecular dynamics (MD) simulations of this peptide, in contact with hydrophobic and hydrophilic self-assembled monolayers (SAMs) and in bulk solution, with in situ measurements of peptide adsorption at these SAM surfaces using quartz crystal microbalance with dissipation monitoring (QCM-D) and spectroscopic measurements. MD simulation has emerged as a powerful tool for the investigation of biomolecules on solid surfaces^11^ as it is able, with appropriate force fields^12^ and sampling methods,^13^ to determine peptide and protein structures on surfaces. These have been used to investigate PAK128-144ox adsorption on self-assembled monolayer surfaces, which offer a route to surfaces with controlled (hydrophobic or hydrophilic) functionality.

## Materials and Methods

### Molecular Dynamics (MD) Simulations

For all simulations, the system contained a single PAK128-144ox peptide, with the initial structure taken from the NMR structure (pdb: 1nim).^14^ Following the biological structure the N-terminus was acylated, with charges on the C-terminus and polarizable residues set appropriate for pH = 7. The surfaces consisted of an alkyl-thiol (RC_11_H_22_SH) self-assembled monolayer, specifically containing either hydrophobic (R = CH_3_) or hydrophilic (R = OH) ligands. The chains were arranged in the √3 × √3 R3 geometry, using structures provided by the Latour research group (https://cecas.clemson.edu/latourlabs/Jmol/Surfaces.html), with 224 (16 × 14) chains in total. For the surface simulations, the simulation box size was 69.2 Å × 70 Å × 100 Å. This box size was sufficient to insulate the peptide from interactions with its periodic images and with the upper wall of the simulation box. To mimic the effect of strong anchoring of the monolayer onto an underlying surface, the terminal sulfur and hydrogen atoms of the chains were fixed.

All systems were constructed using standard Gromacs utilities.^15,16^ For the surface simulations, the protein was initially placed 20 Å from the surface, where the surface was defined as the average *z*-position of the terminal heavy atoms. All systems were solvated and a Na^+^ counterion was added to neutralize the protein. The systems were initially energy-minimized using the steepest descent algorithm followed by short (20 ps) NVT simulations (at 300 K), first with the positions of the heavy atoms in the protein restrained to their initial positions by harmonic potentials (with a force constant of 2.4 kcal mol^–1^ Å^–2^), then without the position restraints. A short (20 ps) NpT-simulation was then performed for the bulk solution.

To describe the inter- and intramolecular interactions the Charmm22* force field^17−19^ was used, along with the CHARMM-TIP3P water model.^20^ Parameters for the alkyl-thiol chains were taken from the CHARMM general force field.^21^

To enhance sampling of protein conformations replica exchange with solute tempering (REST)^22,23^ was employed. This is a variation on replica exchange molecular dynamics, where the temperature varies only for a subset of the system, in this case the protein. The temperature scaling was performed by scaling the protein–protein and protein–solvent interactions by a factor depending on the effective temperature. Specifically, the potential energy was given by
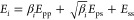
where *E*_pp_, *E*_ps_, and *E*_ss_ are the protein–protein, protein–solvent, and solvent–solvent interaction and the scaling factor β*_i_* = *T*_0_/*T*_*i*_. For all systems, the effective temperature was in the range of 300–440 K, with eight replicas used.^24^ The scaling factors and effective temperatures for the different replicas were 1 (300 K), 0.947 (316.9 K), 0.896 (334.7 K), 0.849 (353.5 K), 0.803 (373.4 K), 0.761 (394.4 K), 0.720 (416.6 K), and 0.682 (440 K). Exchange attempts between neighboring replicas were attempted 1000 time steps (2 ps). Acceptance rates and representative replica trajectories are presented in Table S1 and Figure S1.

Surface simulations were performed in the NVT ensemble with the temperature controlled using a velocity rescaling algorithm,^25^ with a relaxation time of 0.2 ps. Bulk simulations were performed in the NpT ensemble using the Parrinello–Rahman barostat^26^ (relaxation time 2 ps) to control the pressure.

All simulations were performed at a temperature of 300 K and the bulk simulations were performed at a pressure of 1 atm. For the surface simulations, the system was periodic in the *x* and *y* directions. To contain the system in the *z*-direction walls, interaction through the integrated 9-3 LJ potential was used. The bulk simulations were periodic in all directions. All simulations were run for 200 ns, which was sufficient for the number of unique conformations found using cluster analysis to plateau (Figure S2). The peptide secondary structure was also largely consistent after the first 100 ns of the simulations (Figure S3).

A cutoff of 11 Å was used for the van der Waals (VDW) and short-range electrostatic interactions. Long-range electrostatic interactions were evaluated using a particle mesh Ewald sum.^27^ The equations of motion were integrated using a time step of 2 fs, with the LINCS algorithm^28^ used to constrain bond lengths involving hydrogen atoms. Simulations were performed using the Gromacs simulation package^15,16^ (version 2018.4), using the PLUMED library^29^ to implement REST simulations.

### Peptide Sample Preparation

PAK128-144ox was purchased from the Biomolecular Synthesis Facility of the Center for Molecular and Cellular Bioengineering, TU Dresden, Germany, and used without further purification (see Figure S4 for mass spectra of the peptide as provided by the manufacturer). HiPerSolv Chromanorm water for HPLC (VWR Chemicals, France) was used for all aqueous solutions. The peptide was dissolved in 10 mM Na_2_HPO_4_ (Merck KGaA, Germany) at pH 7.4 and concentrations of 0.10 and 2.06 mg/mL for the circular dichroism (CD) spectroscopy and the QCM-D measurements, respectively. The higher concentration used in the QCM-D experiments was chosen to obtain clearly detectable frequency changes within 3 h of adsorption. To avoid bubble formation during the QCM-D measurements, the sample solution was additionally degassed with Ar for 1 h.

### SAM Preparation

Gold-coated quartz crystal sensors were purchased from Fil-Tech, Inc., with a fundamental resonance frequency of 4.95 MHz. Before use, the sensors were immersed in freshly prepared RCA I solution (1:1:5 in volume NH_4_OH/H_2_O_2_/H_2_O) at 75 °C for 30 s. Afterward, the sensor surfaces were washed with water and dried with a stream of ultrapure air. The CH_3_- and OH-terminated SAMs were assembled as previously described^30,31^ by immersion in ethanol (Berkel AHK GmbH & Co. KG, Germany) containing 1 mM 1-octadecanethiol and 11-mercapto-1-undecanol (Sigma-Aldrich, Germany), respectively. After incubation for 24 h, the SAM-coated sensors were rinsed with ethanol and dried with nitrogen.

### QCM-D

Peptide adsorption at the different SAM surfaces was monitored using a Q-Sense E4 (Biolin Scientific, Sweden). The measurements were carried out in static mode at 27 °C. To this end, the flow chamber was flushed with peptide-free buffer for about 60 min at a flow rate of 10 μL/min using a peristaltic pump (IPC4, Ismatech, Germany). After stabilization of the baseline, the peptide solution was injected continuously for 40 min at the same flow rate to completely exchange the cell volume. Then, the pump was stopped and peptide adsorption was monitored under static conditions. After another 185 min, the flow cell was flushed with peptide-free buffer at the same flow rate. In the remainder of the paper, we discuss only the 7th overtone. The data obtained for overtones 3–9 are plotted in Figure S5.

### Polarization-Modulation Infrared Reflection Absorption Spectroscopy (PM-IRRAS)

The secondary structure of the adsorbed peptides on the SAM-modified surfaces of the QCM-D sensors was assessed by PM-IRRAS. To this end, the sensors were removed from the QCM-D flow cells, washed with water to remove residual salt, and dried in a stream of ultrapure air. FTIR spectra of the dry samples were obtained using a Bruker Vertex 70 (Bruker Optics GmbH, Germany) spectrometer with a ZnSe photoelastic modulator (PMA50, Bruker Optics GmbH, Germany). All spectra were recorded with 256 single scans and a resolution of 4 cm^–1^ under an incident angle of 80° with respect to the surface normal, using liquid-nitrogen-cooled mercury cadmium telluride (MCT) detector. The spectrometer and the sample compartment were continuously purged with CO_2_-free dry air (dew point < −30 °C). The secondary structure information of the adsorbed peptides was analyzed by applying Gaussian fits to the second derivative spectra of the amide I bands^32^ (see the Supporting Information). The spectra were background-corrected and fitted using OPUS software (version 7.8, Bruker Optics GmbH, Germany).

### CD Spectroscopy

Circular dichroism (CD) spectra of the peptide (0.1 mg/mL) in 10 mM Na_2_HPO_4_ at pH 7.4 were measured at room temperature in a 1 mm cuvette with a J-815 spectrometer (JASCO) between 185 and 320 nm at a scan rate of 100 nm/min (bandwidth 4 nm). Spectra were averaged over four scans. The secondary structure was estimated with Dichroweb^33,34^ using the method CDSSTR (data set 6).

## Results and Discussion

### PAK128-144ox Adsorption on Hydrophobic and Hydrophilic SAM Surfaces

**Figure 1:**
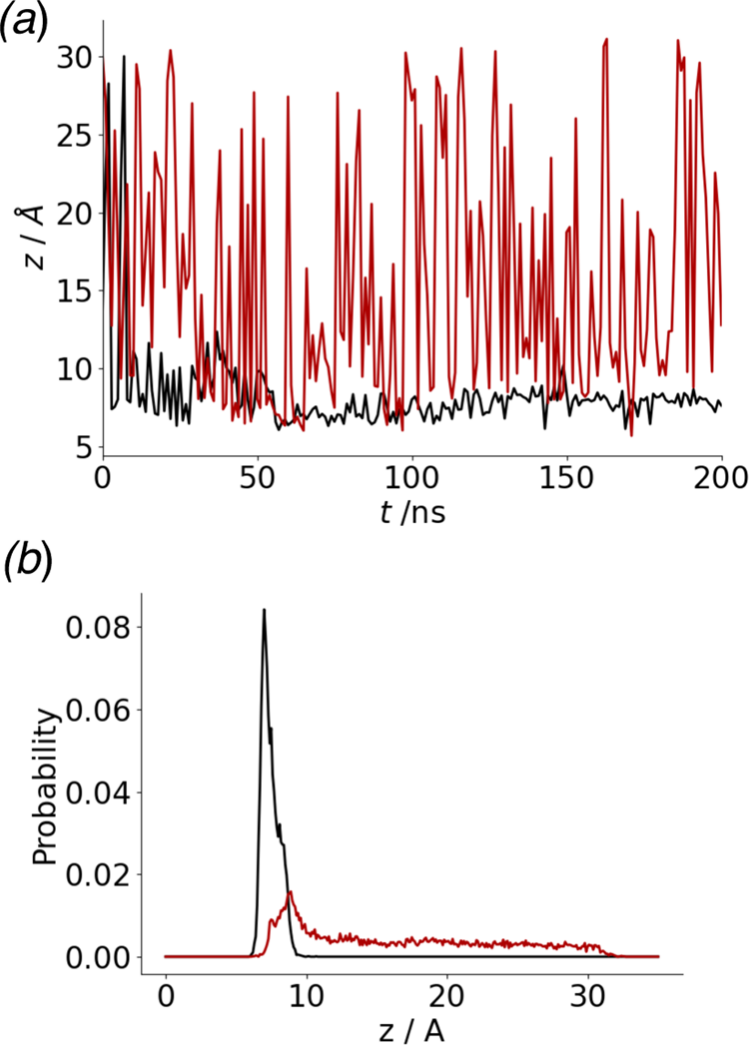
(a) Peptide center-of-mass-surface separation. CH_3_- and OH-terminated surfaces are denoted by black and red lines, respectively. (b) Probability histogram of center-of-mass-surface separation; symbols as in (a).

To investigate the effect of surface hydrophobicity on PAK128-144ox adsorption, we first investigated the peptide in contact with hydrophobic, CH_3_-terminated, and polar, OH-terminated SAMs using MD simulations. On the hydrophobic SAM, PAK128-144ox displays essentially irreversible adsorption. Across the entirety of the simulation, its center of mass is less than 10 Å from the surface (Figure 1a). By contrast, on the hydrophilic OH-terminated SAM, it is significantly less strongly bound, showing transient desorption from the surface. This contrast between hydrophobic and hydrophilic surfaces is consistent with prior simulations of IAPP on these surfaces.^35^

The difference in the adsorption on these two surfaces can be seen by considering the probability histograms of the center-of-mass-surface separation (Figure 1b). On the hydrophobic SAM, this has a sharp peak approximately 8 Å from the surface and with essentially zero probability of it being found farther than 10 Å from the surface. For the polar surface, the probability of finding the peptide in the bulk of the solution is nonzero, consistent with the frequent desorption from the surface. The maximum in the histogram is smaller on hydroxy-terminated SAM, again suggesting weaker binding on this surface. The maximum is also further from the surface compared to the CH_3_-terminated SAM. Notably, there is a shoulder on this peak toward lower separations from the surface, with the closest distance being similar to the closest distance seen for the CH_3_-terminated surface.

**Figure 2:**
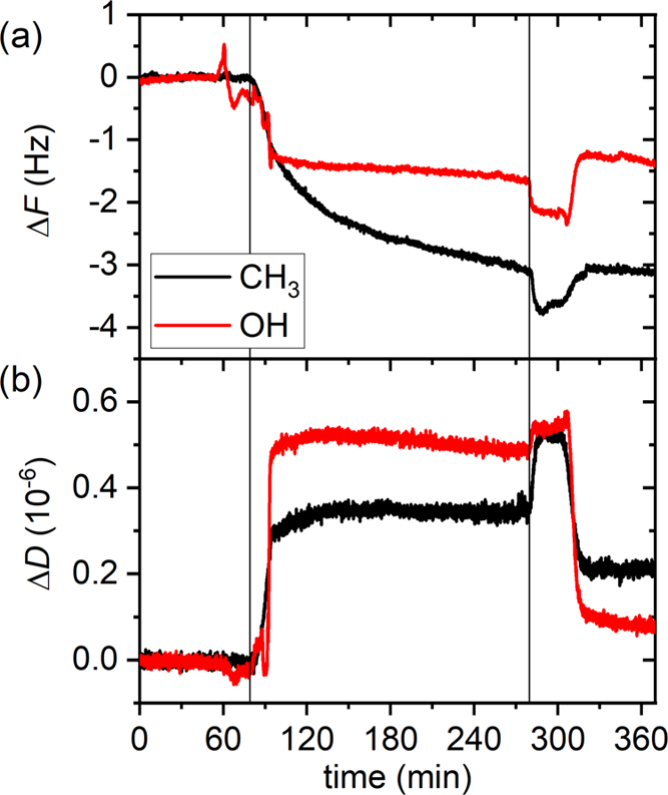
QCM-D results for the adsorption of PAK128-144ox on the CH_3_- and the OH-terminated surface, respectively. (a) Frequency shift Δ*F*. (b) Change in dissipation Δ*D*. Vertical lines indicate the time points of peptide injection and flushing, respectively.

To verify the results of the MD simulations experimentally, we next studied the adsorption of PAK128-144ox on the two different SAM surfaces by QCM-D. QCM-D is a powerful technique for the in situ investigation of biomolecular adsorption at various organic and inorganic surfaces.^36,37^ It enables the monitoring of adsorbed mass via the shift of the resonance frequency Δ*F* of a quartz crystal sensor, while simultaneously providing information about the viscoelasticity of the adsorbed film in the form of the change in energy dissipation Δ*D*. Figure 2a shows the time traces of the frequency shift Δ*F*. For both surfaces, peptide injection results in a sudden decrease in Δ*F*, which is indicative of peptide adsorption. However, for the OH-terminated surface, Δ*F* saturates rather quickly at a comparatively small value of about −1.5 Hz. For the CH_3_-terminated surface, on the other hand, Δ*F* decreases continuously during the time course of the experiment without reaching saturation. The final Δ*F* value recorded before flushing the flow cell was −3 Hz, indicating that the CH_3_-terminated surface adsorbs about twice as much peptide as the OH-terminated one, which is consistent with the weaker attachment seen in the MD simulations. Upon flushing the flow cell with peptide-free buffer, an additional decrease in Δ*F* is observed for both surfaces. This is because these measurements were conducted in static mode so that the peptide-containing solution that remained in the connected tubing during the measurement is pumped through the flow cell before the peptide-free buffer. Since peptide adsorption at the sensor surface over time has resulted in a depletion of peptides in the surface-near region of the bulk solution, this injection of peptide-containing solution and the resulting turbulent flows will lead to a brief concentration spike at the sensor surface and thus additional peptide adsorption. However, peptide adsorption on both SAM surfaces during this short period is fully reversible with the peptides adsorbed at the beginning of the flushing step desorbing again once the peptide concentration in the flow cells has decreased. The Δ*F* value of the CH_3_ surface after flushing is almost identical to the one before flushing, indicating that PAK128-144ox adsorption is virtually irreversible on this surface. For the OH surface on the other hand, some peptide desorption is observed during flushing, leading to a slight increase in Δ*F*.

Figure 2b shows the change in dissipation Δ*D* during adsorption. For both surfaces, peptide adsorption results in a rapid increase in Δ*D*, indicating the formation of a viscoelastic adsorbate film. While the overall increase in Δ*D* is comparatively moderate, as one indeed would expect for such a short peptide, the saturated Δ*D* value is about 50% larger for the OH than for the CH_3_ surface, even though the latter surface adsorbs more peptide (see Figure 2a). This indicates that the PAK128-144ox peptide forms a much more rigid film on the CH_3_ surface than on the OH surface, which is in agreement with the stronger binding seen in the MD simulations (Figure 1). Flushing results in a decreased Δ*D* value for both surfaces, indicating desorption of loosely bound peptides and possibly a compaction of the remaining irreversibly adsorbed peptide film. This decrease in Δ*D* is much stronger for the OH surface, in agreement with the stronger peptide desorption upon flushing observed in Figure 2a. Finally, we would like to point out that no overshooting in the Δ*D* traces in Figure 2b is observed, which implies that there are no structural rearrangements in the adsorbed peptide films on both surfaces. This is in contrast to previous experiments that evaluated the adsorption of this peptide at gold, SiO_2_, and TiO*_x_* surfaces.^10^ For all three surfaces, the Δ*D* traces displayed pronounced spikes in the initial stages of adsorption that were followed by a slow decrease and finally the saturation of the Δ*D* signals. This was explained by residue-driven conformational changes in the peptides during adsorption resulting from electrostatic interactions (SiO_2_ and TiO*_x_*) and covalent bond formation (Au) between peptide and surface, respectively.^10^ For the SAM surfaces studied in the present work, such effects appear to be absent. In summary, the results of the QCM-D experiments are in perfect agreement with the MD simulations.

We have also calculated the adsorbed mass *m* from the stationary Δ*F* values after flushing using the Sauerbrey equation.^38^ For this, all data points over the final 30 min of the experiment have been averaged. As can be seen in Table 1, an adsorbed peptide mass of about 55 and about 24 ng/cm^2^ is obtained at the CH_3_ and the OH surface, respectively. Using the peptide’s estimated hydrodynamic radius^10^ of about 0.8 nm, these mass values translate to surface coverage values Γ of about 0.34 and about 0.15 monolayers (ML), respectively. Using the contact areas between the peptide and the different surface as obtained from the MD simulations yields much larger Γ values of about 0.82 and 0.32 ML for the CH_3_ and the OH surface, respectively, highlighting the important role of surface-specific variations in peptide conformation. While the low Γ value obtained with the OH surface may at first glance be surprising, in particular considering the apparent saturation of adsorption visible in the QCM-D data shown in Figure 2, it is nevertheless comparable in magnitude to those determined previously for PAK128-144ox adsorption at hydrophilic SiO_2_ and TiO*_x_* surfaces based on the hydrodynamic radius of the peptide.^10^ Also in these cases, adsorption saturated well below 1 ML coverage, similar to that observed in the present experiments for the OH surface. Regarding the CH_3_ surface, the coverage of 0.82 derived from the contact area in MD simulations raises some doubts as well since at such a high surface coverage, peptide–peptide interactions will undoubtedly affect the conformation of adsorbed peptides. Therefore, the molecular footprint of a single adsorbed peptide obtained from the MD simulations probably overestimates molecular spreading under such crowded conditions. The real surface coverage will most likely lie between the two extreme estimates of 0.34 and 0.82.

**Table 1:** Adsorbed Peptide Mass *m* and Peptide Surface Coverage Γ at Both SAM Surfaces after Flushing Calculated from the Δ*F* Data Shown in Figure 2a

		Γ (ML)	Γ (ML)
	*m* (ng/cm^2^)	based on hydrodynamic radius^10^	based on MD simulations
CH_3_ surface	54.9 ± 0.6	0.340 ± 0.004	0.817 ± 0.081
OH surface	23.6 ± 1.1	0.146 ± 0.007	0.321 ± 0.046

### PAK128-144ox Structure in Bulk Solution and in Contact with the Different SAM Surfaces

**Figure 3:**
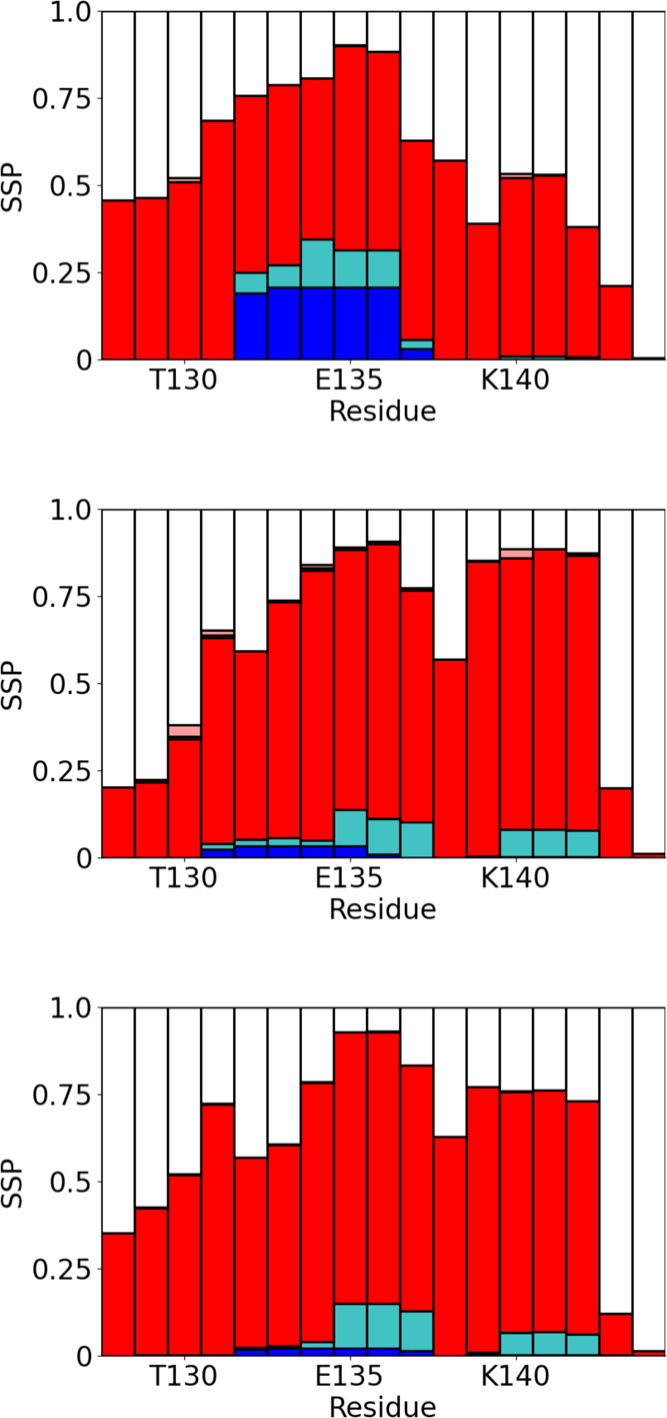
Secondary structure propensities for PAK128-144ox on CH_3_ (top)- and OH (middle)-terminated surfaces and in bulk solution (bottom). White indicates random coil; red, turn; blue, α-helix; turquoise, 3/10-helix; and pink β-bridge.

To investigate the structure of the peptide, the secondary structure has been determined (using the STRIDE algorithm^39^) from simulations of PAK128-144ox on surfaces and in bulk solution (Figure 3). In all cases (CH_3_- and OH-terminated SAMs and bulk solution), the peptide is primarily disordered, with turn or coil being the predominant secondary structure motifs. On the CH_3_-terminated SAM surface, there is a short segment (D132-Q136) that has some tendency toward α-helix formation. This consists of polar and charged residues and is not typically in contact with the surface, suggesting that this is induced by changes in conformation of the surface adsorbed parts of the peptide. The secondary structure on the OH-terminated SAM and in bulk solution is essentially identical, showing a high propensity for turn-formation (Figure 3). The tendency for formation of α-helix is significantly lower than on the CH_3_-terminated SAM. There are some regions (E135-F137 and K140-C142) that show a slight tendency toward the formation of 3/10-helix; however, both stretches of amino acids are shorter than a single helical turn.

Table 2 shows the amount of each secondary structure motif seen in the simulations. Consistent with the secondary structure propensities in Figure 3, β-turn and random coil are the dominant motifs and the amount of α-helix is highest on the hydrophobic surface.

**Table 2:** Secondary Structure Content from MD Simulations

	CH_3_ surface	OH surface	solution
α-helix	0.062 ± 0.121	0.010 ± 0.053	0.008 ± 0.050
3/10-helix	0.031 ± 0.072	0.037 ± 0.074	0.035 ± 0.076
β-turn	0.465 ± 0.227	0.561 ± 0.184	0.571 ± 0.211
β-sheet	0 ± 0.005	0.003 ± 0.032	0 ± 0.002
β-bridge	0.002 ± 0.014	0.014 ± 0.025	0.001 ± 0.010
random coil	0.440 ± 0.175	0.384 ± 0.158	0.384 ± 0.182

**Figure 4:**
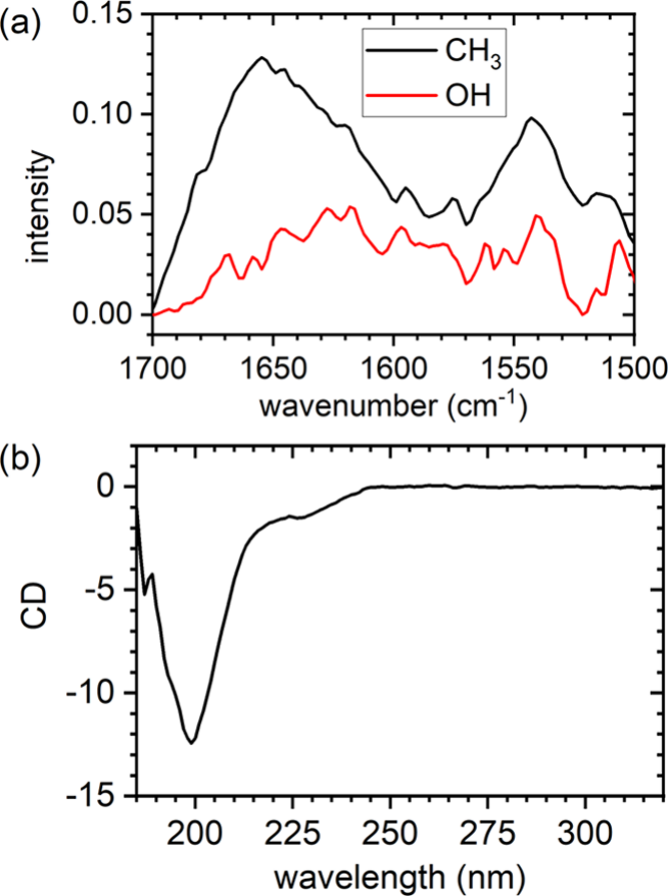
(a) PM-IRRAS results for PAK128-144ox on CH_3_-terminated (black) and OH-terminated (red) surfaces. (b) CD spectrum for PAK128-144ox in solution.

We have additionally attempted to determine the secondary structure elements for the PAK128-144ox peptides irreversibly adsorbed at the different SAMs after the QCM-D measurements using PM-IRRAS. PM-IRRAS is a highly surface-sensitive technique, which enables the quantification of secondary structure elements in the adsorbed peptides by deconvolution of the amide I band.^30,31^Figure 4a shows the combined amide I and amide II region of the recorded spectra. For the CH_3_-terminated surface, both the amide I (around 1650 cm^–1^) and the amide II band (around 1550 cm^–1^) can be clearly identified. For the OH surface, however, both bands have considerably lower intensities and are dominated by noise. This is in line with both the QCM-D measurements and the MD simulations, which showed weaker peptide adsorption on the OH surface. Unfortunately, this low amount of adsorbed peptide on the OH surface prevented us from quantifying the secondary structure elements. The results of the deconvolution of the amide I band of the CH_3_ surface are given in Table 3 (see Figure S6 for fits and Table S2 for fit results). As can be seen, the adsorbed peptides have a rather high β-sheet content (about 39.5%). The second largest contribution stems from β-turns (28.5%), followed by α-helix (16.2%) and random coil (15.9%) contributions. This distribution of secondary structure elements is markedly different from that obtained in the MD simulations (see Table 2). In particular, the MD simulations of the PAK128-144ox peptides in contact with the CH_3_ surface yield β-turns as the most dominant contribution (46.5%), followed by random coil (44.0%). Most strikingly, at this surface, the simulations do not show any β-sheet content.

**Table 3:** Secondary Structure Content from PM-IRRAS and CD Measurements

	CH_3_ surface (PM-IRRAS)	solution (CD)
α-helix	0.162	0.07
β-turn	0.285	0.25
β-sheet	0.395	0.32
random coil	0.159	0.35

To assess whether these strong deviations between experiment and simulation result from inaccuracies in the modeling of the peptide–surface interactions, we have also characterized the structure of the PAK128-144ox peptide in bulk solution using CD spectroscopy. The spectrum is shown in Figure 4b, and the results of the corresponding modeling are summarized in Table 3. In solution, the peptide has a higher random coil content and a lower α-helix content than at the CH_3_ surface. In contrast, both the β-sheet and β-turn contents are rather similar under both conditions. Compared to the MD results in Table 2, there are again discrepancies in the determined secondary structure contributions. This concerns particularly the β-sheet content, which is virtually zero in the MD simulations but contributes ∼32% to the overall secondary structures of the solvated peptide as determined by CD. The discrepancy between experiment and simulation may originate in peptide aggregation, which is not considered in the MD simulations at all. Indeed, in the complete pilin protein, the first four residues of the peptide sequence 128–144 participate in a larger β-sheet assembly as part of the final β-strand.^40^ Therefore, it appears reasonable that the peptides in solution form small oligomeric clusters via the formation of intermolecular β-sheets, which of course may also affect the secondary structure of the parts of the peptide not involved in β-sheet formation. Upon adsorption on the CH_3_ surface, those intermolecular β-sheets do not seem to be disturbed, in contrast to the α-helix and random coil contributions, which change drastically and thus seem to facilitate contact with the surface.

### Which Factors Are Responsible for the Observed Differences in Behavior?

**Figure 5:**
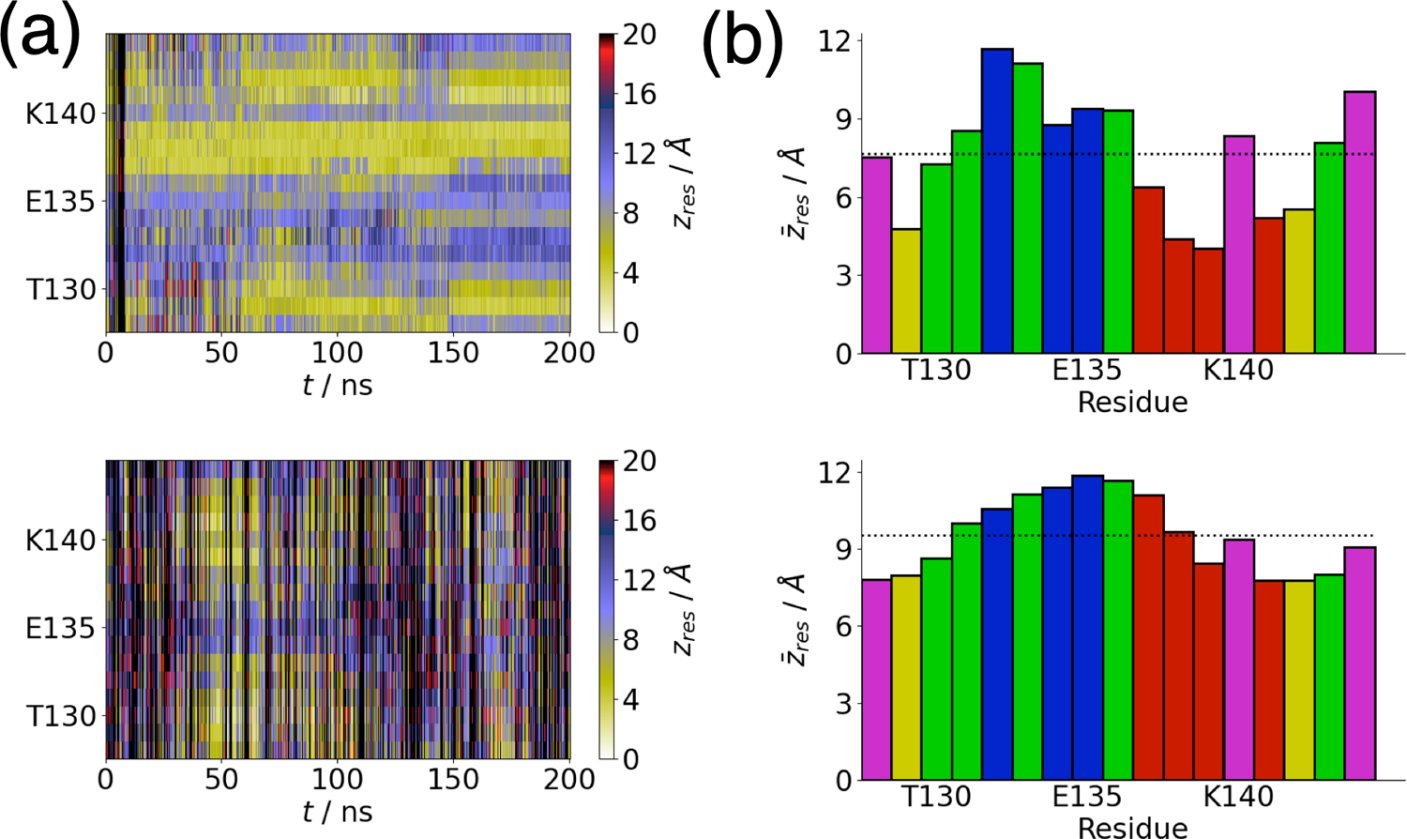
(a) Residue center-of-mass-surface separation for CH_3_ (top)- and OH (bottom)-terminated surfaces. (b) Average residue center-of-mass-surface separations for CH_3_ (top)- and OH (bottom)-terminated surfaces. Red, green, blue, and magenta denote hydrophobic, polar, negatively charged, and positively charged residues, respectively. Cysteine residues are shown in yellow. The dotted line shows the average separation for all residues.

To better understand the different affinities of the peptide to the two investigated surfaces, the primary adsorbing amino acids were identified. Figure 5a shows the residue center-of-mass-surface separations for PAK128-144ox. Consistent with the peptide center-of-mass-surface separation (Figure 1), there are contacts between the peptide and the CH_3_ surface that persist across the simulation. In particular, a group of hydrophobic residues (F137-P139) stays in contact with the surface almost over the entire simulation time. Other sections of the peptide, in particular G141-C142 and C129-T130, show relatively persistent contacts with the surface as well. Due to the disulfide bond between the two cysteine residues, the separation between these two regions and the surface are similar. On the polar OH-terminated surface, transient desorption leads to more variation in the contacts between the peptide and the surface (Figure 5a).

As may be expected, the hydrophobic residues in PAK128-144ox are on average closer to the CH_3_-terminated SAM (Figure 5b). The disulfide bond between C142, which is close to the hydrophobic region of the peptide, constrains the other cysteine (C129) to be close to the surface as well. The polar D132-Q136 region, which shows a slight tendency for helix formation (Figure 3), stays farther from the surface. For the OH-terminated SAM, the average separation of each residue from the surface is higher than for the CH_3_-terminated SAM, consistent with the larger center-of-mass peptide–surface separation (Figure 1). Typically, the regions around the cysteine residues are closest to the surface.

**Figure 6:**
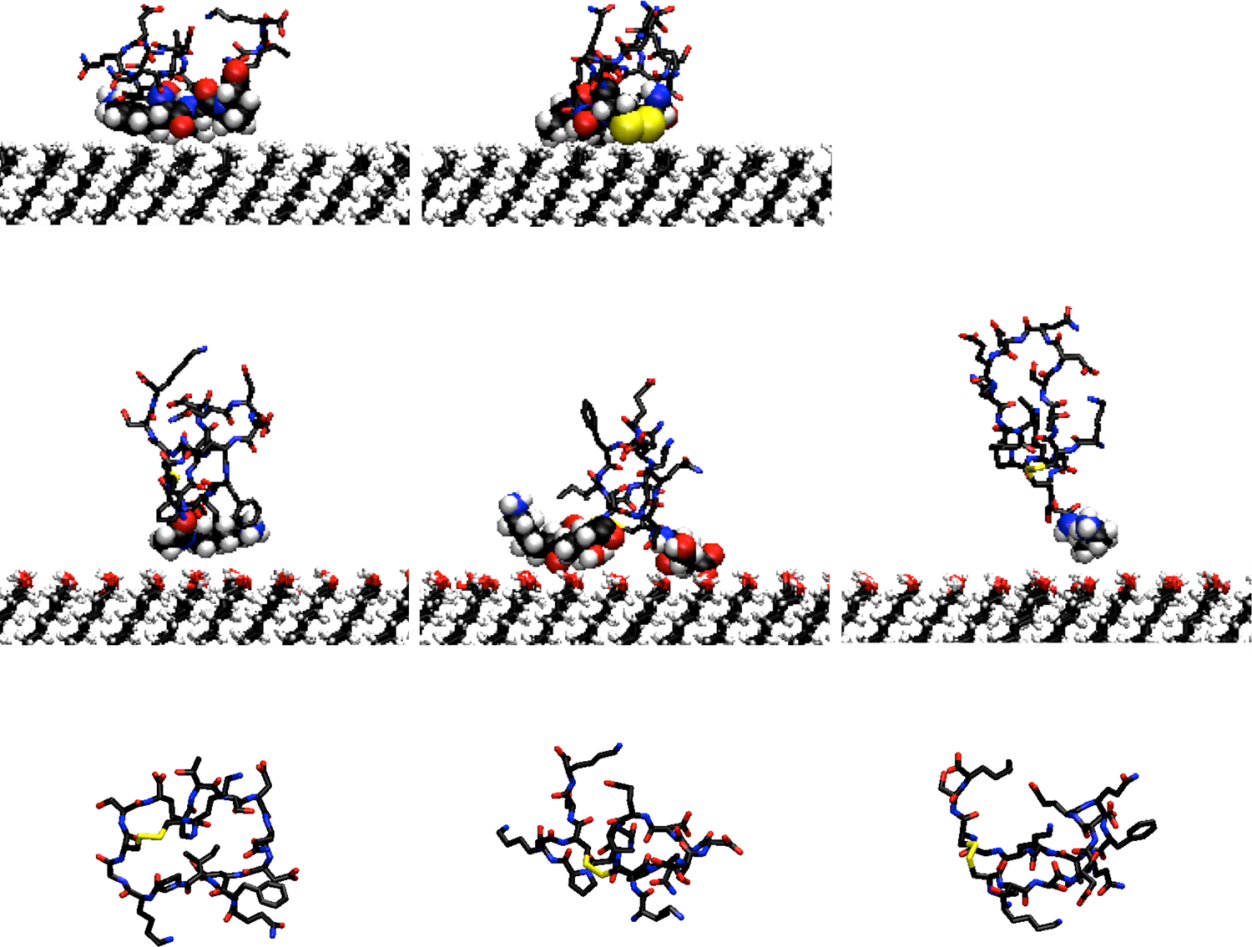
Snapshots showing representative structures from simulations of PAK128-144ox on CH_3_ (top)- and OH (middle)-terminated surfaces and bulk solution (bottom). For surface simulations, residues in contact with the surface are highlighted as spheres.

The different topologies of surface-contacting residues appear causative for the conformation of the adsorbed peptide. Representative snapshots (taken from cluster analysis) of peptide conformations on the two surfaces and in bulk solution are shown in Figure 6. The more persistent contacts with the hydrophobic surface restrict the manifold of PAK128-144ox conformations during the simulation time. On the polar surface, a wider range of conformations is obtained with fewer residues contacting the surface but typically exhibiting adsorption of the C- and N-termini (Figure 5b). The wider distribution of conformations correlates with that of the peptide–surface separations for the OH surface (Figure 1b). Not surprisingly, the conformations adopted in aqueous solution are similar to those on the hydrophilic OH-terminated SAM.

Using the method of Daura et al.,^41^ the peptide conformations were grouped into clusters based on the Calpha RMSD with a cutoff of 2 Å. The restriction of conformational freedom on the CH_3_-terminated SAM is readily appreciated by comparing the number of distinct conformations (Table 4). It is lower on the CH_3_-SAM, due to the pinning of the hydrophobic residues to the surface, whereas it is similar on the OH-terminated SAM and in solution. From these numbers, the conformational entropy (*S*_conf_) was calculated as
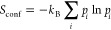
where *p_i_* is the probability of the *i*th conformation and the sum runs over all conformations. As expected, *S*_conf_ is significantly lower on the CH_3_ surface than on the OH-SAM surface or in solution. The lower degree of conformational freedom on the hydrophobic surface may contribute to the higher film rigidity compared to the hydrophilic surface.

**Table 4:** Number of Conformations and Conformational Entropy Determined from Cluster Analysis

	CH_3_-terminated	OH-terminated	solution
no. of conformations	57	254	263
conformational entropy (*k*_B_)	2.05 ± 0.07	3.88 ± 0.23	3.75 ± 0.17

The difference between the conformations on the surfaces also evidences itself through the peptide–surface interaction energy (Table 5). This is higher on the CH_3_-terminated SAM, reflecting the greater number of residues in contact with the surface. For the OH surface, the larger number of adsorbed peptide conformations gives rise to a large variation in the tabulated values. For both surfaces, the VDW interaction is an important driving force for peptide adsorption. The electrostatic contribution is repulsive for the CH_3_ surface. This arises due to repulsion between the positively charged hydrogen atoms on the terminal methyl group and in the hydrophobic residues near the surface. Likewise, the slightly lower conformational entropy disfavors adsorption in this case. However, hydrophobic groups of the peptide, which attach to the CH_3_ surface, also contribute to a gain in entropy by releasing water from the previously exposed interaction sites in the adsorption interface. This source of adsorption-promoting entropy gain is not taken into account but is favorable for the CH_3_-terminated and unfavorable for OH-terminated SAM, respectively.

**Table 5:** Average Peptide–Surface Interaction Energies^a^

	CH_3_-terminated	OH-terminated
*E*_int_ (kJ mol^–1^)	–84 ± 23	–68 ± 115
*E*_int_^VDW^ (kJ mol^–1^)	–107 ± 22	–69 ± 31
*E*_int_^Coul^ (kJ mol^–1^)	24 ± 2	1 ± 97

a Uncertainties estimated using standard deviation.

## Conclusions

Comparing MD simulations with experiments, the affinity and structural impact of the adsorption to hydrophilic or hydrophobic model surfaces of the peptide PAK128-144ox derived from the *P. aeruginosa* type IV pilus was investigated. Both, simulations and experiments, reveal that peptide adsorption is favored on hydrophobic CH_3_-terminated SAMs over “polar”, OH-terminated SAMs. The simulation trajectories show that this is mediated by long-lived contacts between hydrophobic residues and the CH_3_-terminated surface. The weaker adsorption on the polar surface correlates with transient desorption events in the MD simulations and less rigid behavior of the adsorbed peptide film seen in the QCM-D experiments.

The secondary structure of the peptide changes upon adsorption. MD simulations suggest that it is largely disordered, both in bulk solution and on surfaces. The short region (D132-Q136), composed of hydrophilic residues not in contact with the surface, shows a propensity for α-helix formation on the hydrophobic surface. Spectroscopic estimates of secondary structure agree with this tendency seen in the simulations but show also a higher proportion of β-sheet and β-turn compared to the simulations. This increase in ordered secondary structure may indicate aggregation of peptides in the experiments.

The greater number of contacts between the peptide and the CH_3_-terminated SAM surface leads to a stronger interaction, primarily through van der Waals interactions. The strong interaction with the surface also restricts the number of conformations adopted on the hydrophobic surface, compared to the hydrophilic surface, leading to a significant decrease in the conformational entropy.

The combination of the simulation and experimental results allows investigating the driving forces for adsorption. Decomposing the different energetic contributions (Table 5) suggests that on both surfaces, the van der Waals attraction is most prominent, whereas the electrostatic interactions are repulsive but of lower magnitude. The strong adsorption onto CH_3_-terminated SAM mediated by hydrophobic residues (Figure 5) suggests that the hydrophobic effect plays a key role in this case.

While the differences in behavior on the hydrophobic and hydrophilic SAMs seen from the MD simulations and QCM-D measurements are consistent, there is a larger difference in the structure of the peptide derived from simulation and spectroscopy. To address this, future work can include simulation of multiple peptides to investigate their aggregation on surfaces, which the single peptide simulations could not address. From the experimental point of view, harsh pretreatment of the peptides prior to the experiments, for example, with a combination of hexafluoroisopropanol (HFIP) and dimethyl sulfoxide (DMSO), which is frequently used for dissolving amyloid aggregates,^30,31,42^ may provide a better-defined starting solution consisting exclusively of peptide monomers. Even then, however, exposure to an aqueous environment may immediately trigger peptide aggregation. Nevertheless, such an approach may at least enable more detailed experimental investigations of the aggregation mechanism. A study of other, related peptide systems could also be used to determine which aspects of the behavior of PAK128-144ox relate to its specific sequence and which aspects are universal.

## Supporting Information Available

Convergence of REST simulations; peptide characterization; different overtones recorded in the QCM-D measurements; and deconvolution of PM-IRRA spectra (PDF)
