# Supplementary material for: Effect of Surface Hydrophobicity on the Adsorption of a Pilus-Derived Adhesin-like Peptide
Source: Langmuir. 2022 Jul 23;38(30):9257–65. doi: 10.1021/acs.langmuir.2c01016 (PMC9352356; doi:10.1021/acs.langmuir.2c01016)
Supplement: Supplementary file 1 — la2c01016_si_001.pdf [file la2c01016_si_001.pdf]

Effect of surface hydrophobicity on the adsorption of a pilus-derived adhesin-like peptide

Yu Yang,[†] *Jingyuan Huang,*[†] *Daniel Dornbusch*[‡,#] *Guido Grundmeier,*[†] *Karim Fahmy,*[‡,#] *Adrian Keller,*[*,†] *and David L. Cheung*[*,§]

† Paderborn University, Technical and Macromolecular Chemistry, Warburger Str. 100, 33098 Paderborn, Germany.

‡ Helmholtz-Zentrum Dresden-Rossendorf, Institute of Resource Ecology, Biophysics Department, Bautzner Landstrasse 400, 01328 Dresden, Germany.

# Technische Universität Dresden, Center for Molecular and Cellular Bioengineering, 01062 Dresden, Germany.

§ School of Chemistry, National University of Ireland Galway, Galway H91 TK33, Ireland.

Convergence of REST simulations

Table S1. *Acceptance probabilities from REST simulations*

	0↔1	1↔2	2↔3	3↔4	4↔5	5↔6	6↔7
CH_3 SAM	0.255	0.308	0.326	0.301	0.323	0.358	0.342
OH SAM	0.283	0.320	0.342	0.321	0.337	0.371	0.350
Solution	0.292	0.323	0.338	0.332	0.348	0.390	0.370

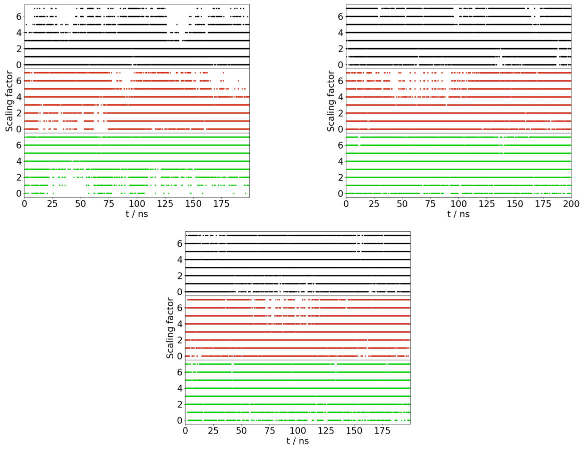

Figure S1. *Plot of scaling factor (i denotes β_i) for PAK128-144ox on the CH_3 SAM (top left), the OH SAM (top right), and bulk solution (bottom). Black, red, and green show replicas with i=0, 4, and 7 at t=0 respectively.*

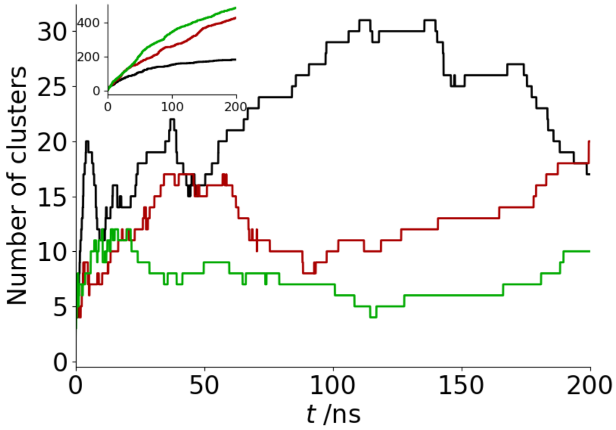

Figure S2. *Change in number of clusters with simulation time for the CH_3 (black) and the OH SAM (red), and bulk solution (green). Main panel shows number of clusters within 3 kcal mol^{-1} of the most common cluster, inset shows total number of clusters.*

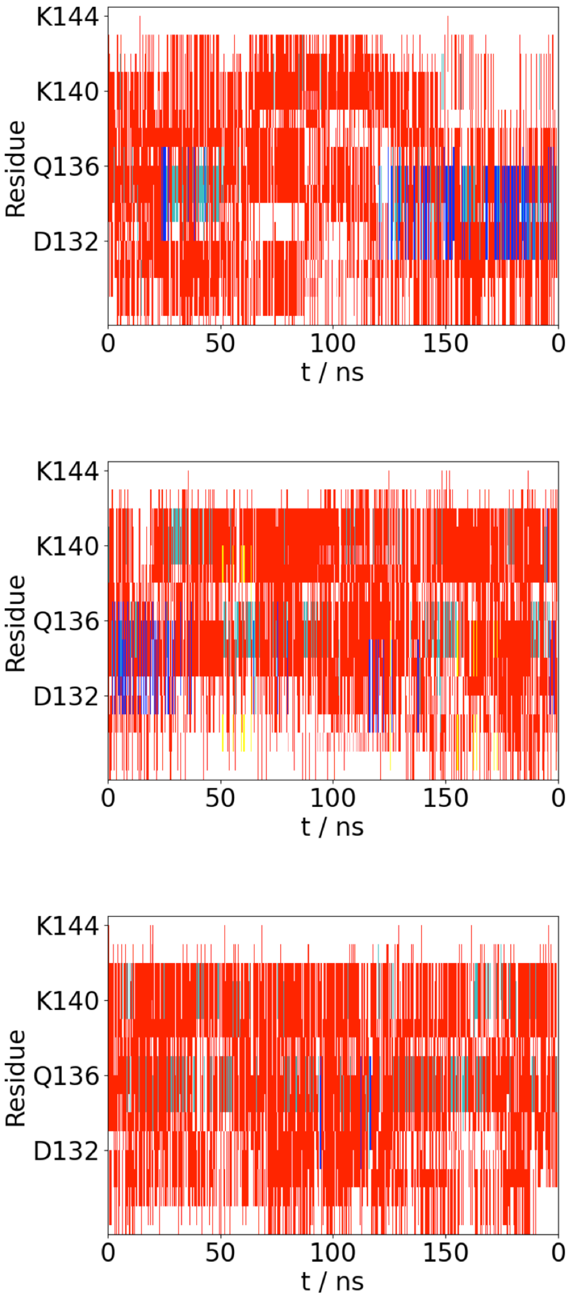

Figure S3. *Plot of peptide secondary for SAMch3 surface (top), SAMoh surface (middle), and bulk solution (bottom). Turn, alpha-helix, 3/10-helix, and beta-strand denoted by red, blue, cyan, and yellow respectively. White areas*

Peptide characterization

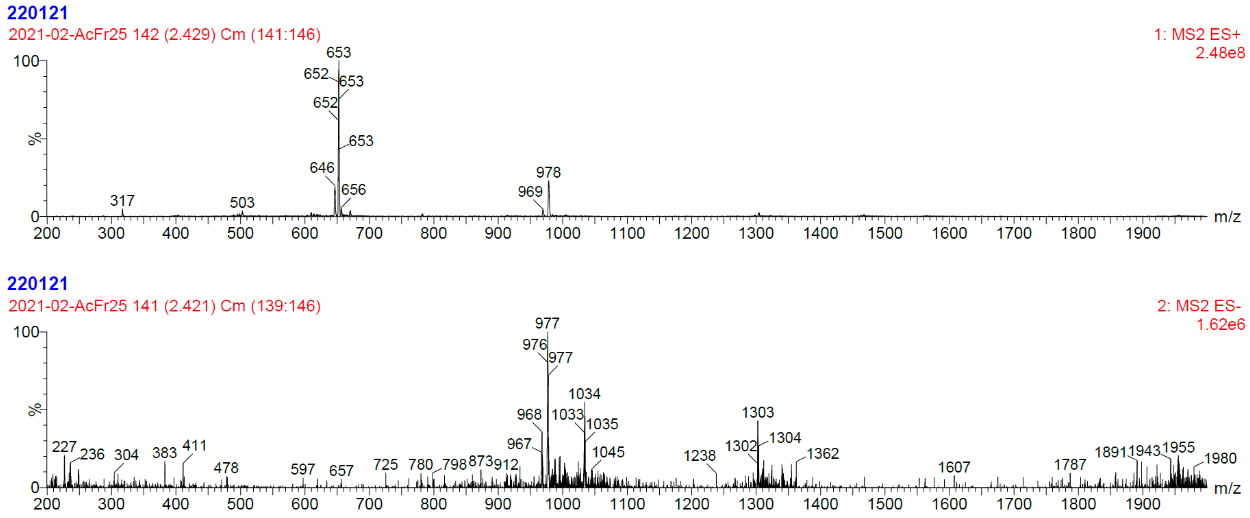

Figure S4. *Mass spectra of the peptide as provided by the manufacturer.*

Different overtones recorded in the QCM-D measurements

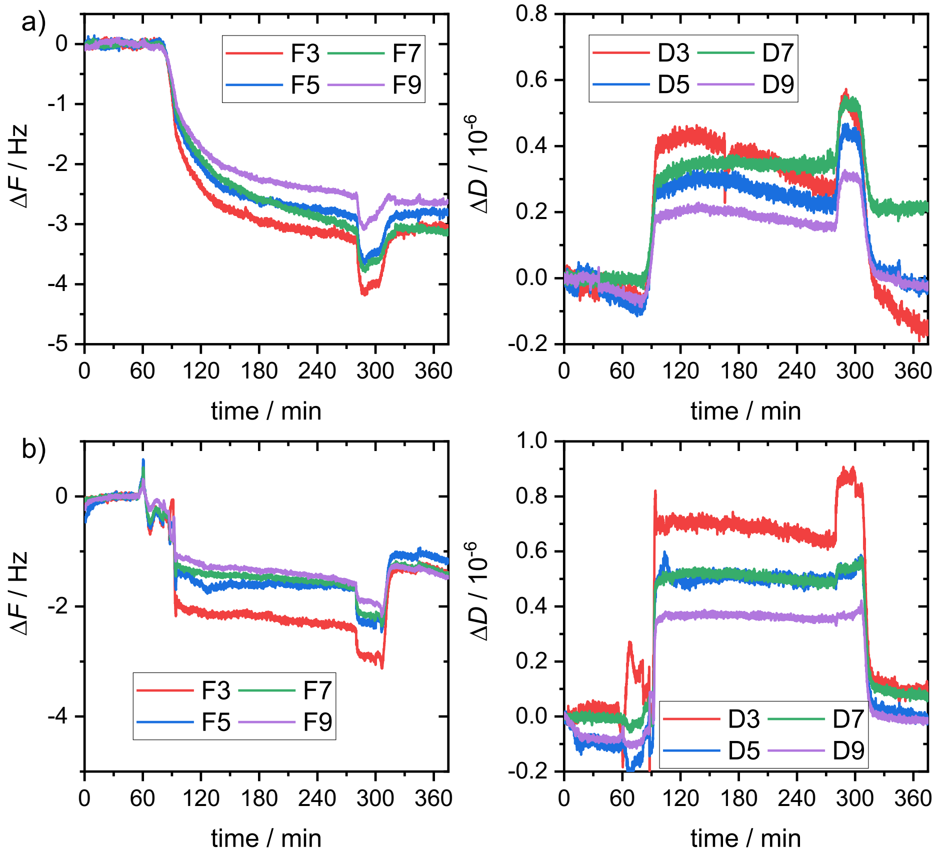

Figure S5. *ΔF and ΔD results (overtones 3 to 9) for PAK128-144ox adsorption on the CH_3 (a) and the OH (b) SAM.*

Deconvolution of PM-IRRA spectra

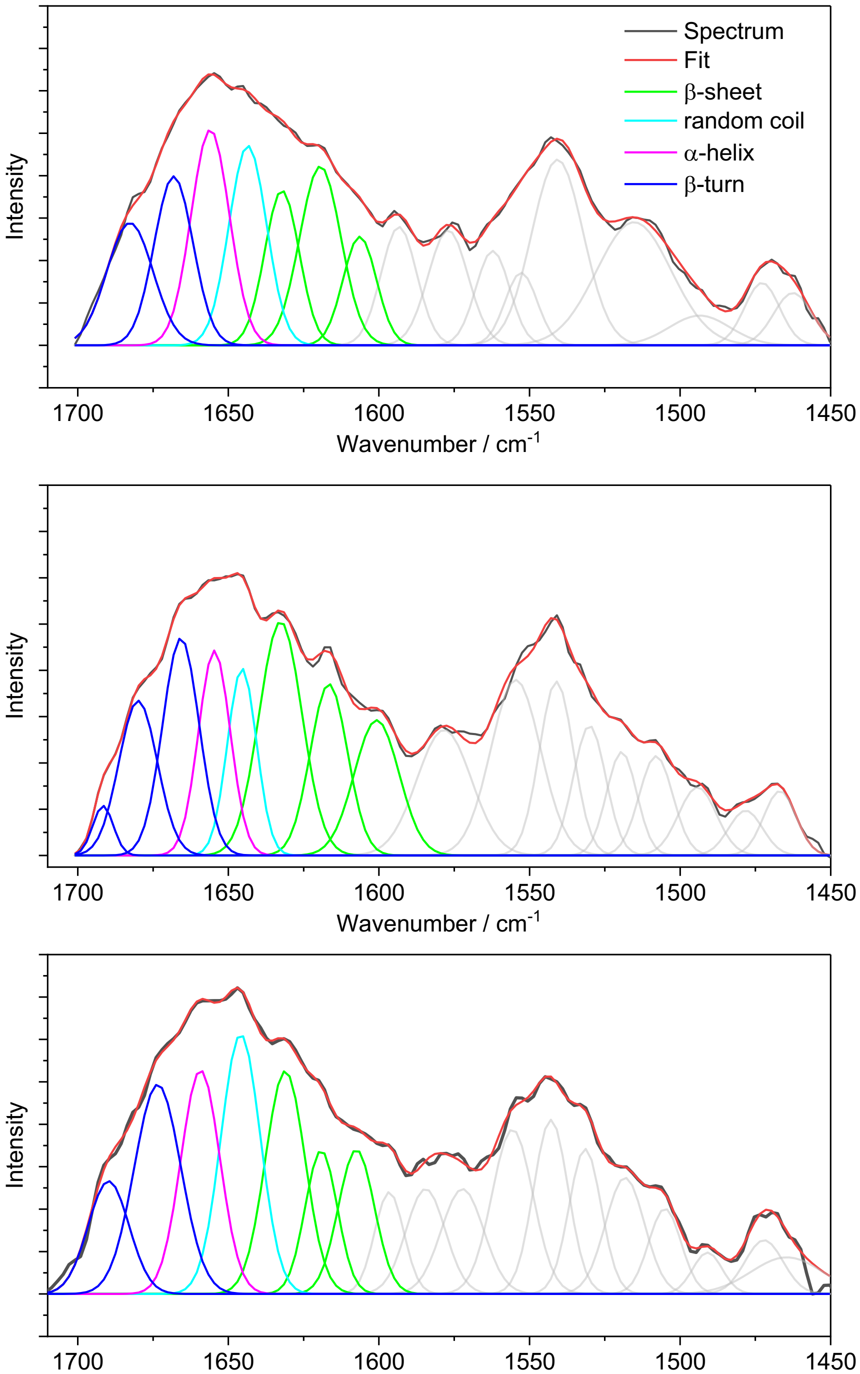

Figure S6. *Deconvolution of the PM-IRRA spectra in the amide region recorded at three different spots on the CH_3 SAM after PAK128-144ox adsorption.*

Table S2. *Secondary structure contents obtained for the deconvolution of the PM-IRRA spectra in Figure S5.*

		Spectrum			
**Peak position / cm^{-1}**	**Assignment**	**# 1**	**# 2**	**# 3**	**Sum average**
1604.9 ± 3.6	β-sheet	0.100	0.123	0.082	0.395
1618.4 ± 1.6	β-sheet	0.089	0.129	0.167	
1632.0 ± 0.8	β-sheet	0.170	0.203	0.120	
1645.0 ± 1.3	random coil	0.197	0.110	0.170	0.159
1656.6 ± 2.4	α-helix	0.168	0.132	0.186	0.162
1667.0 ± 1.7	β-turn	-	0.160	0.148	0.285
1676.7 ± 4.6	β-turn	0.188	0.120	-	
1688.0 ± 4.7	β-turn	0.090	0.021	0.128	
